# Gene Expression Changes in Long-Term In Vitro Human Blood-Brain Barrier Models and Their Dependence on a Transwell Scaffold Material

**DOI:** 10.1155/2017/5740975

**Published:** 2017-11-29

**Authors:** Joel D. Gaston, Lauren L. Bischel, Lisa A. Fitzgerald, Kathleen D. Cusick, Bradley R. Ringeisen, Russell K. Pirlo

**Affiliations:** ^1^American Society for Engineering Education Postdoctoral Fellowship Program, U.S. Naval Research Laboratory, Washington, DC, USA; ^2^Chemistry Division, U.S. Naval Research Laboratory, Washington, DC, USA; ^3^National Research Council Postdoctoral Fellowship Program, U.S. Naval Research Laboratory, Washington, DC, USA; ^4^Defense Advanced Research Projects Agency, Arlington, VA, USA

## Abstract

Disruption of the blood-brain barrier (BBB) is the hallmark of many neurovascular disorders, making it a critically important focus for therapeutic options. However, testing the effects of either drugs or pathological agents is difficult due to the potentially damaging consequences of altering the normal brain microenvironment. Recently, in vitro coculture tissue models have been developed as an alternative to animal testing. Despite low cost, these platforms use synthetic scaffolds which prevent normal barrier architecture, cellular crosstalk, and tissue remodeling. We created a biodegradable electrospun gelatin mat “biopaper” (BP) as a scaffold material for an endothelial/astrocyte coculture model allowing cell-cell contact and crosstalk. To compare the BP and traditional models, we investigated the expression of 27 genes involved in BBB permeability, cellular function, and endothelial junctions at different time points. Gene expression levels demonstrated higher expression of transcripts involved in endothelial junction formation, including TJP2 and CDH5, in the BP model. The traditional model had higher expression of genes associated with extracellular matrix-associated proteins, including SPARC and COL4A1. Overall, the results demonstrate that the BP coculture model is more representative of a healthy BBB state, though both models have advantages that may be useful in disease modeling.

## 1. Introduction

The unique characteristics of the blood-brain barrier (BBB) present a considerable challenge for studying central nervous system (CNS) therapeutics and disease modeling. Lipid-soluble molecules and gases readily diffuse across the BBB while larger, hydrophilic molecules are impeded by endothelial tight junctions [1]. The intrinsic difficulty of crossing the BBB necessitates extensive testing for new drug candidates using relevant in vitro and in vivo models. In vivo model organisms, such as rats, provide valuable information in drug screening assays, particularly with respect to complex effects in the natural CNS environment. However, they are expensive and time consuming, face ethical concerns, require a considerable physical space, and often do no not translate well to human results [2, 3]. One way to overcome the shortcomings of in vivo systems is through in vitro models capable of accurately mimicking human systems. In vitro model systems consisting of cell-based arrays are a valuable tool to screen molecular BBB permeability prior to animal testing.

The most common platform for in vitro BBB testing incorporates multiple cell types cultured on transwell inserts, allowing for separate culture compartments within the same well [1]. The transwell inserts consist of a semiporous polycarbonate or polyethylene terephthalate (PET) membrane. Endothelial cells are grown on one side of the membrane, and astrocytes are grown on the other, creating a simple barrier similar to the natural BBB environment. This basic model setup provides an approximation of the native BBB microenvironment but has several significant shortcomings compared to in vivo models. Membrane material, cell-cell contact, and fluid shear stress are some of the factors poorly addressed by the standard transwell model. Two of these issues, membrane material and cell-cell contact, can be addressed simultaneously by changing the transwell insert membrane material.

Transwell insert membranes are a necessary artifact of barrier culture models, where a substrate is essential for cell seeding, attachment, and monolayer formation. Synthetic barriers, such as polycarbonate or PET, are not readily degraded or remodeled by either endothelial cells or astrocytes, making it an unyielding barrier to “normal” BBB architecture. Without degradation, the membrane also imposes a hard limit on the degree of endothelial-to-astrocyte cell-cell contact. It has been repeatedly demonstrated that proper gene expression and regulation of BBB proteins require direct contact between endothelial cells and astrocytes [4, 5]. Additionally, a monolithic polymer barrier between the cell types prevents proper extracellular matrix (ECM) deposition and basement membrane formation, a factor known to affect BBB permeability [6, 7]. Taken together, these conclusions point to the need for a biodegradable membrane for in vitro models capable of supporting cell growth and remodeling.

Previous research replaced the transwell PET membrane with an electrospun gelatin scaffold, styled “biopaper,” in an endothelial/astrocyte coculture in vitro model [8]. This 21-day study demonstrated that the biopaper coculture models were initially more permeable than standard PET models, at least until day 14. At later time points, the biopaper model was less permeable than the PET, as proven by both transendothelial electrical resistance (TEER) measurements and dextran diffusion across the barrier. Notably, cell morphology was similar in both models, indicating that a deeper look into the gene expression profiles may be necessary to determine differences in transcript expression of essential BBB proteins between the two models.

To date, cellular gene expression profiling of in vitro BBB models has been limited to only a handful of genes relevant to barrier permeability. Recent coculture models typically only assay 5–10 genes, primarily focusing on endothelial cell junction proteins, with studies lasting for several days [9–11]. Furthermore, several recent studies have employed the use of immortalized cell lines or nonhuman animal cell lines. While this data is important for improving BBB models, gene expression profiles obtained from primary human cell coculture models would be invaluable. Furthermore, an extensive comparison of BBB gene expression between models would further illuminate the strengths and weaknesses of each model system.

In order to further characterize the similarities and differences between the biopaper and PET models, we sought to determine differences in gene expression of BBB-related proteins at various time points. We hypothesize that the cell-cell contact and the ability to remodel tissue scaffolding will result in differential gene expression of functionally relevant proteins at the BBB interface, including proteins associated with barrier integrity and overall cell function. Here, we describe the analysis of a 27-gene panel, consisting of genes associated with barrier integrity and endothelial cell or astrocyte function. Prior to the gene expression panel, the suitability of multiple genes to serve as reference genes was examined to ensure an accurate comparison between the models.

## 2. Experimental Procedure

### 2.1. Cell Culture

Primary human astrocytes (HA) and culture media were obtained from ScienCell Research Laboratories (Carlsbad, CA, USA) and maintained according to the manufacturer's instructions. Primary human brain microvascular endothelial cells (HBMEC) and culture media were obtained from Cell Systems (Kirkland, WA, USA) and maintained according to the manufacturer's instructions. Media for both cell types contained 10% FBS. Cells used in all experiments were at passage 1 when seeded.

### 2.2. Biopaper Insert Fabrication

Cell culture inserts with a 15% electrospun gelatin biopaper membrane were fabricated as previously described [8]. Briefly, 10 mL of a gelatin-genipin solution was prepared by dissolving 1.8 grams of type B gelatin from bovine skin (Sigma-Aldrich, Saint Louis, MO, USA) into 70% (vol%) acetic acid (Sigma-Aldrich) in distilled water. The gelatin solution was magnetically stirred for 60 minutes before a solution consisting of 0.05 grams of genipin (Wako Chemicals, Richmond, VA, USA) dissolved in 0.5 mL of ethanol and 1 mL of 1X phosphate-buffered saline (PBS) was added to the solution. The homogenous solution was then transferred to a 20 mL plastic syringe (Thermo Fisher Scientific, Rockwood, TN, USA) with a 22-gauge stainless steel blunt-ended needle (Jensen Global, Santa Barbara, CA, USA). The spinning solution was stored between 24 and 72 hours until electrospinning.

Gelatin nanofiber biopapers were electrospun using a Matsusada Precision AC100-240V high-voltage power supply (Kusatsu city, Shiga, Japan) and a New Era Pump NE-300 syringe pump (Farmingdale, NY, USA). The power supply electrode was connected to the stainless steel blunt-ended needle and positioned 15 cm from a grounded circular stainless steel plate (10 cm diameter) covered with nonstick aluminum foil. The syringe pump was set to a flow rate of 5 *μ*L/min, and a voltage of 15 kV was applied to the electrode for a total of 30 minutes to produce each electrospun mat, designated “biopapers.” Electrospun biopapers were placed in a Sanplatec Dry Keeper desiccator (Kita-ku, Osaka, Japan) for at least 24 hours.

Biopapers were cut to size and placed over the opening of hanging cell culture inserts (Millipore, Billerica, MA, USA) from which the factory PET membrane was removed. The edges of the biopaper were heat-sealed to the cell culture insert. The excess biopaper around the edges was removed. To cross-link the biopapers, the inserts were placed in 24-well plates, submerged in a 0.5 mL solution consisting of 5% (*w*/*v*) genipin dissolved in ethanol, and incubated for 7 days at 37°C. Inserts were rinsed four times with sterile water and stored in water at 4°C until needed, but not longer than 7 days. As demonstrated in previous work [8], this protocol produces biopapers with an overall thickness of 4.5 ± 1.5 *μ*m. Prior to use, inserts were sterilized by placing under UV light for at least 20 minutes.

### 2.3. In Vitro BBB Model

In vitro bilayer human blood-brain barrier (BBB) models were prepared both with standard PET cell culture inserts (1 *μ*m pore size) and with electrospun biopaper membranes. First, inserts were inverted and placed onto the cover of a 24-well plate. Fifty microliters of HA medium containing 25,000 HAs was added to the bottom side of each insert. The well plate was used to cover the inserts which were placed in an incubator at 37°C for 2 hours to allow for cell attachment. After 2 hours, the plates were righted and 1 mL of HA medium was added to the bottom of each well. The following day, 500 *μ*L of HBMEC medium containing 25,000 HBMECs was added to the top of the cell culture insert. Only the first passage of cells was used to minimize phenotypic changes from experiment to experiment. Medium formulation used for the experiment was identical to that used for cell culture prior to seeding. The medium was changed every 2-3 days.

Inserts were harvested to analyze transcriptional differences via reverse transcription quantitative polymerase chain reaction (RT-qPCR) to compare models between cells grown on the PET or biopaper. Using forceps, each membrane was pulled from the hanging plastic insert and fully submerged in the Allprotect Tissue Reagent (Qiagen, Germantown, MD) in a 1.5 mL tube. Inserts were harvested after 3, 7, 14, 21, and 28 days with six inserts collected for both PET and biopaper membranes at each time point. Each biopaper and PET insert was seeded with a different cell preparation, resulting in *n* = 6 biological replicates. Samples in Allprotect were kept frozen at −20°C until processed for RT-qPCR.

### 2.4. Fluorescent Immunohistochemistry

At day 21, three samples from both the biopaper model and PET model were set aside for fluorescent immunohistochemistry analysis. Prior to staining, all samples were removed from the transwell insert and placed on a glass slide. Samples were fixed in 4% paraformaldehyde for 10 minute at room temperature, followed by three five-minute washes with phosphate-buffered saline (PBS). Following fixation, all samples were permeabilized with a 0.25% Triton X-100 PBS solution for 10 minutes at room temperature. Samples were washed three times with PBS and incubated with a blocking solution of 1% bovine serum albumin (BSA) and 22 mg/mL glycine in PBS containing 0.1% Tween 20 for 30 minutes at room temperature. After blocking, samples were incubated with primary antibodies for platelet adhesion cellular adhesion molecule 1 (PECAM-1, mouse, Thermo Fisher 37-0700) and glial fibrillary acidic protein (GFAP, chicken, Thermo Fisher PA1-10004) in PBS containing 0.1% Tween 20 for 1 hour at room temperature. Dilution of both primary antibodies was 1 : 50. Following primary antibody incubation, samples were washed with PBS three times, five minutes each, and incubated with a secondary antibody solution for 1 hour at room temperature in the dark. The secondary antibody solution consisted of goat anti-mouse (SAB 4600082, Sigma) and goat anti-chicken (SAB 4600465, Sigma) in PBS containing 1% BSA and 0.1% Tween 20. Three final washes with PBS were performed on the samples, after which the Vectashield antifade mounting reagent was applied (H-1400, Vector Labs, Burlingame, CA) and coverslips were mounted. Samples were imaged the next day on a Nikon Eclipse Ti confocal microscope using NIS-Elements software (NIS AR 4.10).

### 2.5. Reverse Transcription Quantitative Polymerase Chain Reaction (RT-qPCR)

Prior to RNA extraction, the samples were centrifuged at 3200 rpm for 5 minutes and washed twice with 0.5 mL 1X PBS. Membranes were removed from the original 1.5 mL collection tubes with sterile forceps and transferred to sterile 2 mL tubes containing 350 mL Buffer RLT (Qiagen, Germantown, MD). A 5 mm stainless steel bead (Qiagen, Germantown, MD) was added to the tube followed by lysis on a Tissuelyser LT (Qiagen, Germantown, MD) at 50 Hz for 2 minutes. Samples were spun at 15,000 rpm for 3 minutes, and the supernatant was transferred to a new 2 mL tube. Total RNA was purified with the RNeasy Plus Mini Kit (Qiagen, Germantown, MD) using the QIAcube (Qiagen, Germantown, MD), an automated sample processing technology, according to the manufacturer's instructions. The DNase digestion was extended to 1 hour. Total RNA concentration and purity were quantified using a Nanodrop 2000 spectrophotometer (Thermo Fisher Scientific, Waltham, MA). Total RNA was converted to complementary DNA (cDNA) using the RT^2^ First Strand Kit (Qiagen, Germantown, MD) protocol with 45 ng total RNA per reaction. Each genomic DNA elimination mix contained 45 ng total RNA and 2 *μ*L Buffer GE and was brought to a final volume of 10 *μ*L with RNase-free water. Reactions were incubated for 5 min at 42°C, whereupon they were placed on ice and the components of the reverse transcription reaction added according to the manufacturer's instructions, in a total volume of 10 *μ*L. Reactions were incubated at 42°C for 15 min, followed by 5 min at 95°C. A total of 91 *μ*L RNase-free water was added to each 20 *μ*L cDNA reaction and stored at −20°C until further use. It should be noted that extracted RNA from each sample continues the contributions from both endothelial cells and astrocytes. Due to the experimental setup, it was impossible to separate endothelial cell RNA and astrocyte RNA.

### 2.6. Housekeeping Gene Arrays

A human housekeeping profiler RT^2^ PCR Array was used to assess a suite of genes for use as potential reference genes. This array consisted of 12 housekeeping genes in a 96-well format (PAHS-000Z, Qiagen, Germantown, MD). The stability of the genes was assessed on 28 samples representative of both biopaper and monocultures of all cell types. Each gene was assessed with two different final template (cDNA) concentrations: 1.6 ng and 0.1 ng. Due to very low total RNA yields of monocultures, reference gene assays were not performed with these samples. The RT^2^ SYBR Green Master Mix (Qiagen, Germantown, MD) was used for all reference gene assays. For all assays, each reaction consisted of 12.5 *μ*L 2x RT^2^ SYBR Green Mastermix, 2 *μ*L cDNA, and 10.5 *μ*L nuclease-free water. The following protocol was used for all assays: an initial 10-minute incubation at 95°C followed by 40 cycles of 95°C for 15 seconds and 60°C for 1 minute. A single reaction was performed for each gene on each sample. Reactions were recorded and analyzed using the Applied Biosystems ViiA™ 7 System software. The PCR efficiency of each gene was assessed via LinRegPCR [12]. The expression stability of all candidate reference genes across all samples was assessed using the BestKeeper v. 1 software program. [13]

### 2.7. Custom RT^2^ PCR Array for RT-qPCR

A custom profiler RT^2^ PCR Array Format C was designed with 27 RT^2^ Primer Assays, 2 housekeeping genes (NONO and RPLP0), and 3 controls (human genomic DNA contamination, reverse transcription, and positive PCR control) (SABiosciences). The 32 genes were arranged in triplicate within a 96-well PCR array. The array was prepared using the published protocol. Briefly, for one sample with analysis on 32 targets, 450 *μ*L 2x RT^2^ SYBR Green Mastermix was added to 34 *μ*L cDNA synthesis reaction and 416 *μ*L RNase-free water and mixed well. Twenty-five microliters of the PCR component mix was added to each well of the custom array, and the plate was sealed with MicroAmp Optical Adhesive Film. After a brief spin, the array was placed in a Fast 96-well block of a ViiA 7 Real-Time PCR System (Applied Biosystems, Foster City, CA). The cycling conditions were an initial 10-minute incubation at 95°C followed by 40 cycles of 95°C for 15 seconds and 60°C for 1 minute. The Applied Biosystems ViiA 7 software was used to record and analyze all reactions. The fluorescence threshold was manually adjusted for each gene assay based on visual inspection of fluorescence in the log phase.

### 2.8. Data Analysis

The expression level of each gene within a given time point was compared between the biopaper and PET coculture models using the ΔC_T_ method [14]. All samples used NONO as a reference gene, having been validated as the best housekeeping gene of the ones tested. Briefly, the ΔC_T_ value of each gene was obtained by subtracting the C_T_ value of the reference gene (NONO) from the target gene. This value was linearized through a log2 transformation (i.e., 2^−ΔCT^). Within each time point, the final 2^−ΔCT^ expression values for the biopaper and PET models were compared using two-tailed Student's *t*-test, with results considered significant at *p* < 0.05. This procedure was repeated for all genes at all time points. Notably, two biopaper replicates and two PET replicates from the day 7 time point were removed from all data analyses, as well as one PET replicate from day 3. These samples were removed due to either complete lack of expression of GFAP and AQP4 (known astrocyte-specific markers) or significantly increased C_T_ values (Table S1) [15, 16]. It is our belief that the astrocytes in these samples died and such they are not representative of the astrocyte/endothelial model of this experiment.

## 3. Results

### 3.1. Validation of Housekeeping Genes

All tested reference genes possessed similar PCR efficiencies (Table 1). At the low (0.1 ng) cDNA concentration, NONO was the most stable gene (standard deviation (SD) = 1.39) across all sample types, though the stability of all genes was above the recommended cut-off value of 1.0 (Table 1). At the high (1.6 ng) cDNA concentration, the SD of all genes decreased, indicating greater stability. As with the low template concentration, PP1H (SD = 0.77), NONO (SD = 0.84), and GUSB (SD = 0.84) were the most stable of all genes tested, followed by RPLP0 and HSP90AB1 (both with SD = 1.02). NONO was chosen as the housekeeping gene for the custom RT^2^ qPCR array, due to having high stability at both high and low concentrations.

### 3.2. Fluorescent Immunohistochemistry

**Figure 1:**
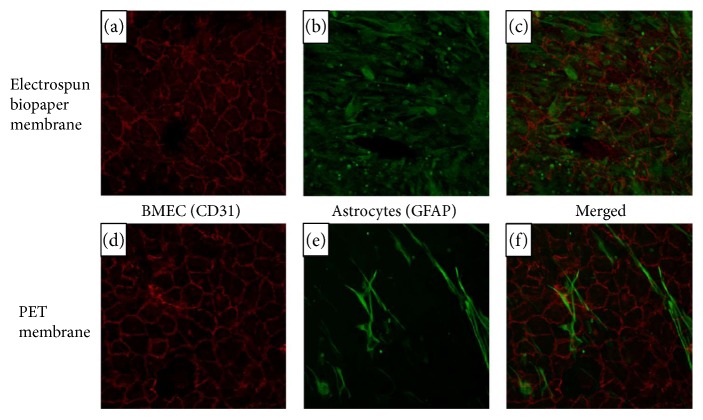
Fluorescent immunohistochemistry of biopaper and PET membranes. PECAM-1 (red) is present at BMEC junctions in both models (a, d). GFAP (green) is distributed throughout the astrocyte, showing similar morphology across membrane materials (b, e). Merged images are shown in (c) and (f).

At day 21, fluorescent staining clearly showed the presence of the BMEC marker PECAM-1 and the astrocyte marker GFAP (Figure 1). The cobblestone pattern typical of an endothelial monolayer can also be readily observed, as well as an elongated astrocyte morphology. Morphology of BMEC and astrocyte grown on the biopaper or PET is very similar, indicating that the membrane materials may not have had a discernable effect on cellular morphology.

### 3.3. Gene Array Data

Little to no expression was observed at any time point in either the biopaper or PET coculture systems for genes gap junction *β*4 (GJB4), gap junction *β*6 (GJB6), tight junction protein 3 (TJP3), and claudin 3 (CLDN3) (Table 2). As these genes had no expression values, statistical analysis could not be performed.

At day 3, a significant difference in gene expression between coculture systems was observed for six genes (Table 2). Three of these six genes had higher expression in biopaper cocultures, including gamma-glutamyltransferase 5 (GGT5, *p* = 0.002), tight junction protein 2 (TJP2, *p* < 0.001), and cadherin-5 (CDH5, *p* = 0.02). Conversely, cells grown on PET had significantly higher expression for laminin subunit *α*1 (LAMA1, *p* = 0.02), platelet-derived growth factor receptor *β* (PDGFRB, *p* < 0.001), and leukemia inhibitory factor (LIF, *p* = 0.018) at this time point.

Significant differences in gene expression were observed for nine genes at day 7. Cocultures grown on the biopaper had significantly higher expression of laminin subunit *α*4 (LAMA4, *p* = 0.018), platelet-derived growth factor *β* (PDGFB, *p* < 0.001), GGT5 (*p* = 0.004), CDH5 (*p* < 0.001), TJP2 (*p* = 0.001), and platelet endothelial cell adhesion molecule (PECAM1, *p* < 0.001). Compared to PET cocultures, significantly lower expression was observed for secreted protein that is acidic and rich in cysteine (SPARC, *p* = 0.003), collagen IV *α*1 (COL4A1, *p* = 0.003), and PDGFRB (*p* = 0.004).

Unlike previous time points, most of the eleven genes with differential expression at day 14 had higher expression in PET cocultures compared to the biopaper cocultures. Specifically, PET cultures were observed to have significantly higher expression of SPARC (*p* = 0.022), laminin subunit *α*2 (LAMA2, *p* = 0.006), LIF (*p* = 0.045), fibroblast growth factor 2 (FGF2, *p* = 0.029), claudin 12 (CLDN12, *p* = 0.003), tight junction protein 1 (TJP1, *p* = 0.008), gap junction *α*1 protein (GJA1, *p* = 0.049), and vascular cell adhesion molecule 1 (VCAM1, *p* < 0.001). Only PDGFB (*p* = 0.004), TJP2 (*p* = 0.03), and CDH5 (*p* = 0.001) had higher expression on the biopaper at this time point.

Day 21 had three genes with statistically significant expression differences between the two coculture systems. Cells grown on the biopaper had significantly higher expression of PDGFB (*p* = 0.007) and claudin 1 (CLDN1, *p* = 0.047) but significantly lower expression of VCAM1 (*p* = 0.017) at this time point.

The final time point, day 28, had six genes with differential expression. Higher expression of LAMA4 (*p* = 0.021), PDGFB (*p* < 0.001), PDGFRB (*p* = 0.01), CDH5 (*p* < 0.001), TJP2 (*p* = 0.035), and PECAM1 (*p* = 0.048) was observed in biopaper cocultures compared to PET cocultures. Notably, none of the assayed genes had higher expression in PET cultures at this time point.

**Figure 2:**
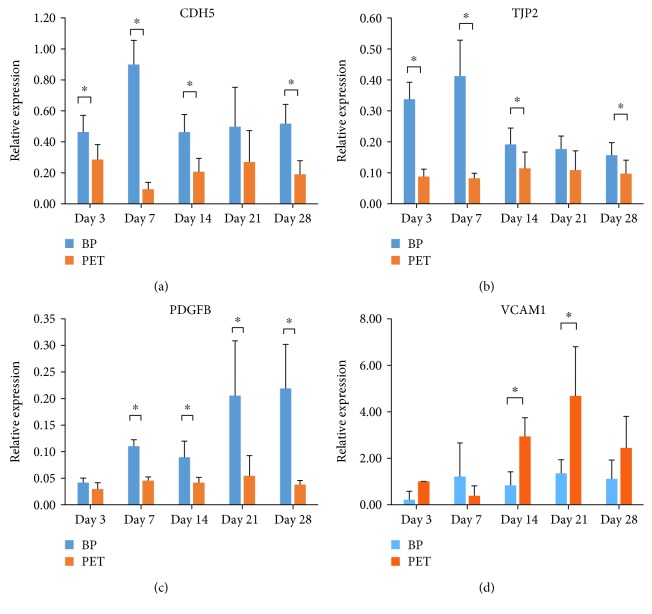
Differences in gene expression across time points between the biopaper and PET for (a) CDH5, (b), TJP2, (c) PDGFB, and (d) VCAM1. In all graphs, gene expression is blue for the biopaper model and orange for the PET model. ^∗^*p* < 0.05.

Four genes, CDH5, TJP2, PDGFB, and VCAM1, with significant roles in BBB maintenance and permeability had significant differences in expression between models across multiple time points (Figure 2). CDH5 and TJP2 were significantly more highly expressed in the biopaper model at all time points except day 14 (Figures 2(a) and 2(b), resp.). PDGFB had significantly higher expression for all time points after day 3 (Figure 2(c)), and VCAM1 was more highly expressed in the PET coculture model for days 14 and 21 only (Figure 2(d)).

## 4. Discussion

In order to accurately model the BBB in vitro, cellular gene expression must be limited to only genes normally expressed at the BBB in vivo. In order to confirm this for both the biopaper and PET models, GJB4 and GJB6 were included on the array as negative controls. GJB4 and GJB6 code for gap junction proteins connexin 30.3 and connexin 30, respectively. Connexin 30.3 expression has been demonstrated in non-BBB endothelial tissue, but there is currently no record of it being expressed by either astrocytes or brain microvascular endothelial cells. Thus, the absent expression of GJB4 in this study is consistent with literature findings, confirming that neither model expresses barrier proteins not present at the BBB. Unlike connexin 30.3, connexin 30 is a crucial component of gap junctions between astrocytes and plays an important role in synaptic activity [17]. However, astrocytic expression is tightly controlled by neurons [18], which were not present in either the biopaper or PET models. As such, the lack of GJB6 expression is readily explained and expected by the lack of cellular signaling from neurons within the culture. Taken together, these findings demonstrate that both coculture models do not display gene expression not associated with the BBB, due to either constitutive lack of expression or the requirement of additional cellular cues (i.e., neuron presence).

Expression of only endothelial, not epithelial, tight junction genes was confirmed though the use of negative controls TJP3 and CLDN3. TJP3, which codes for the tight junction protein zonal occludens-3 (ZO-3), is primarily expressed at epithelial tight junctions [19]. To date, there is no evidence of it being expressed at the BBB, consistent with findings in both the PET and biopaper models. Similarly, CLDN3 is expressed at very low levels in brain tissue but instead has significantly higher expression at epithelial barriers [20]. The results obtained from these negative controls demonstrate that neither the biopaper nor PET model systems expressed proteins associated with epithelial, not endothelial, barriers.

Expression levels of all endothelial tight junction transmembrane proteins assayed were equal between the biopaper and PET coculture models at all time points, with the exception of CLDN12 and CLDN1. Interestingly, expression of occludin (OCLN) and claudin-5 (CLDN5), the primary proteins comprising tight junctions [21], was not significantly different between model systems. Low expression of occludin is a hallmark of several disrupted BBB disease states [22], and claudin-5 plays a critical role in tight junction size-selective permeability [23]. This finding suggests that the membrane material, whether PET or gelatin biopaper, does not have a drastic effect on expression of high-abundance tight junction proteins. It also demonstrates for the first time that endothelial cells grown on an electrospun biopaper model do not have lower expression of these tight junction proteins than those grown on traditional models. This is true for all assayed endothelial transmembrane proteins except for claudin-12 (CLDN12) and claudin-1 (CLDN1). CLDN12, like CLDN5, is located at tight junctions, though it is primarily associated with epithelial cells. To date, there is little record of CLDN12 expression in BBB-derived endothelial cells. Recent evidence does suggest that CLDN12 is expressed by neural stem cells and decreases upon differentiation [24]. The presence of quantifiable CLDN12 expression in both PET and biopaper model systems may be explained by some neural stem cells being present in the initial primary cell populations. This finding points to the limitation of this study; commercially sourced primary cells are purified but may still have some residual cells capable of expansion and model alteration. This limitation is also supported by the commercial entities themselves, as the endothelial cells are guaranteed to be >95% pure, not necessarily 100%. Claudin-1 (CLDN1) forms the tight junction barrier in conjunction with CLDN3, CLDN5, and OCLN and was more highly expressed in the biopaper model at one time point. Though there is some evidence that increased expression of CLDN1 reduces BBB permeability to blood-borne molecules [25], it is probable that the observed level of expression did not have a quantifiable effect on barrier integrity differences between the models. The difference in expression, though significant, was slight (*p* = 0.047) and did not persist for more than one time point. Additionally, one PET sample was considerably lower than the rest, potentially causing the statistical difference observed. Future studies should incorporate more samples, in order to obtain datasets with lower standard deviation between samples. Overall, these findings indicate that extracellular endothelial cell-cell adhesion and physical molecule impedance at tight junctions are most likely similar between the two coculture models.

Higher expression of CDH5 at multiple time points indicates adherens junctions may result in decreased permeability in the biopaper model. VE-cadherin, encoded by CDH5, is the primary integral membrane protein of endothelial adherens junctions [26]. It plays a multifunctional role within the adherens junction, aiding in endothelial cell survival, stabilizing blood vessel assembly, and altering vascular permeability [26]. Low levels of CDH5 expression are indicative of increased barrier permeability, a method exploited by some viruses [27]. The higher expression levels of CDH5 observed in the biopaper model may signify more or tighter adherens junctions than those observed in the PET model, indicative of higher barrier integrity or regulation.

Despite the similarities in expression level of tight junction transmembrane proteins between the biopaper and PET cocultures, the zonal occludens accessory protein family had different expression profiles. TJP1, coding for protein zonal occludens-1 (ZO-1), had similar expression in the biopaper and PET models at all time points except day 14, where the observed expression is significantly higher in the PET model. Formation of both tight and adherens junctions requires proper localization and assembly of ZO-1 scaffolding prior to recruitment of transmembrane proteins such as occludin or claudins [28]. The similar level of expression indicates that endothelial cells grown on biopaper or PET most likely assemble tight and adherens junctions along similar timeframes. Interestingly, protein zonal occludens-2 (ZO-2, expressed from TJP2) is significantly more highly expressed in the biopaper model at all time points except day 21. Unlike ZO-1, ZO-2 is found exclusively at tight junctions, where it plays a significant role in junction assembly [29]. Though not yet fully elucidated, it also appears to have a direct correlation with BBB integrity; multiple studies have shown a decrease in ZO-2 expression preceding increased BBB permeability [30–32]. As such, the biopaper coculture model would be expected to be less permeable to molecular diffusion at all time points, a finding not supported by previous research [8]. Future studies should look to clarify the discrepancy between mRNA levels and diffusion data by assaying subcellular localization and abundance of ZO-2.

Secreted by endothelial cells [33], PDGFB plays a critical role in BBB formation and maintenance, acting as a chemoattractant for pericytes [34] and altering gene expression. The expression levels of PDGFB increased over time in the biopaper model only, with levels remaining relatively constant in the PET model. This pattern is reflected in expression levels between the two models at each time point; no significant difference was observed at the day 3 time point, but PDGFB was more highly expressed in the biopaper model at all later time points. Failure of endothelial cells to recruit pericytes results in mechanical instability, eventually leading to microaneurysm [35] and embryonic lethality [36]. Though pericytes were not present in either the PET or biopaper models, higher PDGFB expression in the biopaper model may create a better gradient for putative pericyte chemoattraction. Furthermore, the elevated expression level of PDGFB over time in the biopaper model indicates it would be better for a long-term BBB model, as continued expression of PDGFB is necessary to maintain barrier integrity [37]. The addition of pericytes to both PET and biopaper models may further elucidate functional differences between models due to PDGFB expression.

Four genes were expressed at significantly higher levels at multiple time points in the PET model. One of these genes, vascular cell adhesion molecule 1 (VCAM1), is correlated with increased BBB permeability. VCAM1 plays a critical role in neutrophil migration into the CNS by opening pores in the BBB, allowing cells to cross the barrier [38]. Endothelial cells normally express VCAM1 at low levels but dramatically upregulate expression in response to inflammatory conditions [39]. The increased expression and accompanying change in endothelial morphology are exploited by several virus families for BBB penetration, as demonstrated both in vitro with dengue virus [40] and in vivo with Venezuelan equine encephalitis virus [41]. The result that VCAM1 was significantly higher in the PET model compared to the biopaper model at days 14 and 21 is relevant from a disease pathology standpoint, as it may be useful for inflammatory modeling, further supporting the suggestion that the PET membrane is more indicative of an inflamed model. Like VCAM1, LIF is expressed at significantly higher levels in the inflamed central nervous system [42]. Though most research on the effects of LIF on the CNS has been focused on the spinal cord, multiple studies have shown it to be a potent proinflammatory cytokine [43, 44]. The significantly higher levels present in the PET model suggest the model may be more indicative of an “inflamed” state, potentially useful for testing the effects of a treatment on inflammation. On the other hand, the biopaper model may be more relevant for investigating the progression of inflammation and disease states from a “healthy” baseline.

It is particularly interesting that previous TEER testing does not correlate well with gene expression results obtained in the current study. Prior analysis demonstrated that endothelial/astrocyte bilayers grown on the biopaper had average TEER values of 12 *Ω*cm^2^ at day 4 and 18 Ωcm^2^ at day 9, significantly lower than the average TEER values of 14 Ωcm^2^ and 27 Ωcm^2^ recorded for PET bilayers at the same time points [8]. The expression data, particularly from barrier genes such as TJP2 and CDH5, would seem to indicate that the biopaper model should have a higher TEER value than the PET model at these early time points. However, there could be multiple reasons for the discrepancy between gene expression data and functional outcomes. One explanation is that, as previously noted, BBB permeability is a complex process that requires many proteins working it in concert. It is possible that higher gene expression of other accessory proteins is required in order to achieve higher TEER levels early. Another explanation is that there may be a delay between gene expression and enough protein accumulation to effect functional changes. It is possible that a “threshold” level of TJP2 or CDH5 is necessary to upregulate other cellular changes leading to a tighter barrier, as is observed for other genes [45]. Further research is needed to elucidate the timeline and correlation between BBB gene expression and functional barrier permeability.

## 5. Conclusions

In vitro coculture models of the BBB comprised of endothelial cells and astrocytes respond differently to transwell membranes of either electrospun gelatin biopapers or PET. Of the 27 genes analyzed, 4 had little to no expression, 5 had no significant difference in gene expression levels between the biopaper and PET inserts, 10 had significantly higher expression in at least one time point in the PET model, and 7 had significantly higher expression in at least one time point for the biopaper model. The fact that less than a third of the genes tested had similar expression between insert materials means there was a significant difference between these two models. While both are valid models, our studies validate the use of different membranes based on the experiments to be tested. Both model systems have similar expression of integral membrane proteins at tight junctions but differ in expression of accessory proteins such as ZO-2. The difference in expression of several key inflammatory genes such as VCAM1 and LIF indicates that the PET model would be optimal for studying the effects of various therapies on BBB in the inflamed state. Conversely, the biopaper model has higher expression of genes associated with a “tighter” BBB for culture times of at least 28 days, thus validating the use of the biopaper model for long-term studies. Future experiments should focus on a subset of these tested genes and correlate mRNA level with protein abundance. Furthermore, the application of the PET model for studying the inflamed BBB should be further investigated.

## Figures and Tables

**Table 1:** Housekeeping gene PCR efficiency and stability.

Target gene	PCR E	0.1 ng stability (SD)	1.6 ng stability (SD)
ACTB	1.91	5.70	1.14
B2M	1.90	2.54	1.08
GUSB	1.93		0.84
GAPDH	1.80	2.25	
HPRT1	1.94	1.43	1.23
HSP90AB1	1.95	1.46	1.02
LDHA	1.94	1.84	
NONO	1.96	1.39	0.84
PGK1	1.92	4.11	1.21
PP1H	1.93		0.77
RPLP0	1.92	4.30	1.02
TFRC	1.91	3.12	1.06

**Table 2:** Significant differences in gene expression between the biopaper and PET coculture systems at each time point. Genes with significantly higher expression on the biopaper and PET are shown in italic and bold formats, respectively. Genes with no expression on the biopaper or PET at any time are presented with dashes.

		Day 3	Day 7	Day 14	Day 21	Day 28
Extracellular matrix-associated proteins	SPARC		**p** = 0.003	**p** = 0.022		
	COL4A1		**p** = 0.003			
	LAMA1	**p** = 0.02				
	LAMA2			**p** = 0.006		
	LAMA4		*p* = 0.018			*p* = 0.018
	AGRN					

Functional astrocyte proteins	GFAP					
	AQP4					

Membrane transporters and soluble factors	PDGFB		*p* < 0.001	*p* = 0.004	*p* = 0.007	*p* < 0.001
	PDGFRB	**p** < 0.001	**p** = 0.004			*p* = 0.01
	GGT5	*p* = 0.002	*p* = 0.004			
	LIF	**p** = 0.018		**p** = 0.045		
	FGF2			**p** = 0.029		

Endothelial tight junction transmembrane proteins	OCLN					
	CLDN1				*p* = 0.047	
	CLDN3	—	—	—	—	—
	CLDN5					
	CLDN12			**p** = 0.003		

Endothelial adherens junction transmembrane proteins	CDH5	*p* = 0.02	*p* < 0.001	*p* = 0.001		*p* < 0.001

Endothelial junction accessory proteins	TJP1			**p** = 0.008		
	TJP2	*p* < 0.001	*p* = 0.001	*p* = 0.03		*p* < 0.001
	TJP3	—	—	—	—	—

Gap junction proteins	GJA1			**p** = 0.049		
	GJB4	—	—	—	—	—
	GJB6	—	—	—	—	—

Cell adhesion proteins	VCAM1			**p** < 0.001	**p** = 0.02	
	PECAM1		*p* < 0.001			*p* = 0.048

## Supplementary Materials

Supplementary files Absent gene expression in 4 samples for both AQP4 and GFAP. The highlighted samples were removed from all analyses.
