# Supplementary material for: Gene Expression Changes in Long-Term In Vitro Human Blood-Brain Barrier Models and Their Dependence on a Transwell Scaffold Material
Source: J Healthc Eng. 2017 Nov 29;2017:5740975. doi: 10.1155/2017/5740975 (PMC5727720; doi:10.1155/2017/5740975)
Supplement: Supplementary files — Absent gene expression in 4 samples for both AQP4 and GFAP. The highlighted samples were removed from all analyses. [file 5740975.f1.pdf]

| Time point | Sample ID | Gene target | Ct |
|---|---|---|---|
| Day 3 | PET-1 | AQP | 26.890 |
| Day 3 | PET-2 | AQP | 26.242 |
| Day 3 | PET-3 | AQP | 29.431 |
| Day 3 | PET-4 | AQP | Undetermined |
| Day 3 | PET-5 | AQP | 28.018 |
| Day 3 | PET-6 | AQP | 25.643 |
| Day 3 | PET-1 | GFAP | 24.457 |
| Day 3 | PET-2 | GFAP | 22.673 |
| Day 3 | PET-3 | GFAP | 25.393 |
| Day 3 | PET-4 | GFAP | 32.599 |
| Day 3 | PET-5 | GFAP | 24.892 |
| Day 3 | PET-6 | GFAP | 23.415 |
| Day 7 | BP-1 | GFAP | Undetermined |
| Day 7 | BP-2 | GFAP | Undetermined |
| Day 7 | BP-3 | GFAP | 25.533 |
| Day 7 | BP-4 | GFAP | 28.843 |
| Day 7 | BP-5 | GFAP | 23.216 |
| Day 7 | BP-6 | GFAP | 25.083 |
| Day 7 | PET-1 | GFAP | Undetermined |
| Day 7 | PET-2 | GFAP | Undetermined |
| Day 7 | PET-3 | GFAP | 23.146 |
| Day 7 | PET-4 | GFAP | 23.262 |
| Day 7 | PET-5 | GFAP | 23.386 |
| Day 7 | PET-6 | GFAP | 24.292 |
| Day 7 | BP-1 | AQP4 | Undetermined |
| Day 7 | BP-2 | AQP4 | Undetermined |
| Day 7 | BP-3 | AQP4 | 30.653 |
| Day 7 | BP-4 | AQP4 | 30.291 |
| Day 7 | BP-5 | AQP4 | 23.342 |
| Day 7 | BP-6 | AQP4 | 26.972 |
| Day 7 | PET-1 | AQP4 | Undetermined |
| Day 7 | PET-2 | AQP4 | 31.232 |
| Day 7 | PET-3 | AQP4 | 24.729 |
| Day 7 | PET-4 | AQP4 | 24.585 |
| Day 7 | PET-5 | AQP4 | 26.218 |
| Day 7 | PET-6 | AQP4 | 25.785 |

Table S1. Absent gene expression in 4 samples for both AQP4 and GFAP. The highlighted samples were removed from all analyses.
